# Determinants of chronic energy deficiency among adults living with HIV in Shebel Berenta District, East Gojjam, Amhara region, North West Ethiopia, 2017: case control study

**DOI:** 10.1186/s13104-019-4443-5

**Published:** 2019-07-17

**Authors:** Masreshaw Tadele, Mulugeta Tesfa, Grimay Tsegaye, Habtamu Temesgen, Nakachew Mekonnen Alamirew

**Affiliations:** 1 Shebel Berenta Woreda Health office, Shebel Berenta Woreda, Yeduha, Ethiopia; 2 Department of Public Health, College of Health Sciences, Debre Markos University, Debre Markos, Ethiopia; 3 Department of Nutrition and Food Science, College of Health Sciences, Debre Markos University, Debre Markos, Ethiopia

**Keywords:** Chronic energy deficiency, Shebel Berenta District, HIV/AIDS

## Abstract

**Objective:**

To assess determinants of chronic energy deficiency in adults living with HIV in Shebel Berenta District Anti-Retroviral Therapy (ART) site health centers, East Gojjam, Amhara region, Ethiopia, 2017. An institutional based unmatched case control study design was employed and simple random sampling was used to select the desired sample size for both cases and controls. Data were entered to Epi-Data 3.1, exported to SPSS version 20 for analysis. Binary logistic regression was used to identify the determinants of chronic energy malnutrition among Human Immune Deficiency Virus positive adult patients.

**Results:**

A total of 473 (118 cases and 355 controls) People Living with Human Immune Deficiency Virus (PLHIV) adult patients were participated. PLHIV who started ART at world health organization (WHO) clinical stage I (AOR: 0.285, CI 0.10, 0.81), rural residents (AOR: 0.38, CI 0.17, 0.83), had family size ≤ 3 (AOR: 0.114, CI 0.03, 0.48) and changed their feeding style (AOR: 0.075, CI 0.038, 0.150) decreased the risk of chronic energy deficiency. However, the baseline CD4 cell < 200/mm^3^ (AOR: 13.398; CI 4.83, 37.19), monthly family income ≤ 500 Ethiopia Birr (AOR: 6.9, CI 1.07, 44.62) and interrupted treatment (AOR: 2.28, CI 1.02, 5.09) were increasing the risk of chronic energy deficiency. Therefore; the government and partners should focus on the above determinants to improve the nutritional status of the clients.

## Introduction

Human immune deficiency virus/acquired immune deficiency syndrome (HIV/AIDS) related complications and under nutrition have a complex interaction and often coexist. Malnutrition has a synergistic effect on the immune system and HIV affects nutritional status [1–3].

Globally, out of the 36.9 million people living with HIV/ AIDS, 25.8 million (70%) live in sub Saharan Africa. The large number of deaths (39 million) and serious infections (75.9 million) occurred due to HIV related diseases [4, 5]. In Ethiopia, the national prevalence of HIV/AIDS is 1.2%. With heterogeneity in urban and rural areas, the Amhara regional State accounts 1.32% of prevalence rate [6]. The HIV/AIDS epidemic often occurs in a population with high prevalence of malnutrition. Protein energy malnutrition or chronic energy deficiency is identified as the most common form of adult malnutrition in sub Saharan Africa correlates with HIV/AIDS patients [7].

HIV contributes to malnutrition and can cause decreased caloric intake; increase loss of nutrients and increase use of energy. Tuberculosis (TB) and diarrhea, which are common in HIV infection have the severe nutritional consequence that usually aggravates appetite loss, weight loss and wasting [7, 8].

The mere increment in coverage of ART services does not prevent the impact of malnutrition. Hence, nutritional therapy and support should be integrated at the time of ART initiation [9]. PLHIV are more likely to become malnourished because of reduced food intake emanating from loss of appetite, poor absorption of nutrients that occurred due to chronic diarrhea, viral intestinal damage, increased demand associated with viral replication and opportunistic infection. The consequences of malnutrition and HIV have a vicious cycle (National Guide line for HIV/AIDS and nutrition in Ethiopia).

Recognizing chronic energy deficiency is important because it may predict disease progression, risk of morbidity and mortality. Good nutrition may help to prolong the period of time for HIV infection and the onset of opportunistic infections. Even though different studies indicate that PLHIV are prone to malnutrition, there is no documented studies about determinants of chronic energy deficiency in the study area. Thus, this study aimed to assess determinants of chronic energy deficiency in adults living with HIV in Shebel Berenta District Anti-Retroviral Therapy site health centers, East Gojjam, Amhara region, Ethiopia, 2017.

## Main texts

### Methods

#### Study design, periods and setting

Institutional based un-matched case control study was conducted at Shebel District, North West Ethiopia from March 15 to April 25, 2017. The District is located in East Gojjam zone, Amhara region state, North West Ethiopia and about 127 km away from Debre Markos, zonal town and 387 km from Bahir Dar, the regional city. It has two ART site health centers, which provide treatment, care and preventive health services for about 772 HIV positive patients.

#### Participants

The source populations for this study were all PLHIVs who were enrolled in the ART clinic for chronic HIV/AIDS treatment, care and support services and who are 18 years old and above. BMI greater than or equal to 18.5 kg/m^2^ for controls and BMI less than 18.5 kg/m^2^ for cases were the study population. Clients who are seriously ill and pregnant HIV positive women were excluded.

#### Sample size determination and sampling techniques

The sample size was determined using statistical software EPI- Info version 7.0 by taking 95% CI and 90% power. Nutritional support in PLHIV was taken as a factor and risk of exposure to 13.22% of the controls and it was 28.16% of cases. By taking 12% non-response rate, the final sample size was found to be 479. We use 3: 1 ratio for control and cases which becomes 329 and 120 respectively [8].

First, to identify the cases and controls, the nutritional status was determined using a BMI before data collection during follow- up periods. After identifying the Cases and controls, we prepared the sampling using the clients’ unique ART numbers. Computer generated simple random sampling technique was employed to select the desired samples of case and control groups from the listed sample frame.

#### Data collection tools and procedures

Interviewer administered structured questionnaire was used to collect required data. Firstly, it was prepared in English language and later translated into local language, Amharic. Standard anthropometric measurements (weight and Height measurement scales) were used to assess nutritional status using weight and height. During weight measurement, the clients were asked to remove heavy clothes and the weight scale was at zero level before each measurement. Weight was recorded to the nearest 0.1 kg. Weight scales were calibrated daily for accuracy. At the time of height measurement, clients were asked to remove their shoes and stand erect on heels, buttock, shoulder blades, and occipital bone against the wall and eyes facing straight forward.

#### Study variables

*Chronic energy deficiency* (case /control) were the dependent variable. *Socio demographic variables* (Sex, Age, Marital status, Income, Education, Occupation, Religion, and Residence), *Medical and morbidity variables* (Diarrhea, Current clinical condition, vomiting, Eating problems, Opportunistic infections, Intestinal parasite, Loss of appetite), *Nutritional variables* (Nutritional counseling, Nutritional support, feeding style change), *Clinic, treatment and accessibility variables* (CD4 level, Side effect of drugs, WHO clinical staging, Adherence, Drug regimen type, Duration of ART, Distance from a health center) were independent variables.

#### Data quality control

One-day training was given for the data collectors (Four comprehensive ART trained health care workers) and supervisors. The data collection was done under daily close supervision and were checked for completeness every day. Before starting any measurement, scales were calibrated daily. Pretest was done on 5% of the sample at Bichena Health center (which is out of the study area) before data collection to check the appropriateness of the questionnaires.

#### Data processing and analysis

Data were entered into EpiData version 3.1 statistical software and exported to the Statistical Package for Social Science (SPSS) version 20.0 for analysis. Descriptive statistics were employed to give a brief description. The binary logistic regression model was fitted to identify which variables were determinants for chronic energy deficiency. Variables, which showed association with bivariable analysis at p-value of less than 0.2 was entered into multivariable analysis. Finally, variables whose p value less than 0.05 at 95% CI were identified as statistically significant determinants for chronic energy deficiency.

### Results

#### Socio-demographic characteristics of the respondents

A total of 473 adult HIV clients who were on ART with the response rate of 98.7% participated in the study. Among 473 participants, 355 were controls and 118 cases**.** Out of 473 participants, 255 were females, 200 (56%) controls and 55 (46%) cases. The mean age of participants was 39.27 (SD ± 9. 082) years. Two hundred seventy-four (77%) controls and 80 (70%) cases were living in rural areas. 248 (70%) controls and 85 (72%) cases were married. Two hundred thirty-one (49%) participants can read and write while two hundred forty one (69%) of controls and 78 (66%) cases were farmers (Table 1).

**Table 1:** Socio demographic and economic Characteristics of people living with HIV/AIDS, Shebel Berenta District, East Gojjam, Amhara region, North West Ethiopia, 2017

Variables	Controls (N = 355)		Cases (N = 118)	
	No	%	No	%
Age groups				
18–27 years	50	14	6	5
28–47 years	234	65.9	83	70
48–60 years	71	20	29	24
Sex of participant				
Female	200	56	55	46
Male	155	43	63	53
Religion of participants				
Orthodox	223	62	97	82
Muslim	132	37	21	17
Marital status				
Single	45	12	8	6
Married	248	70	85	72
Divorced	36	10	10	8
Widowed	26	7	15	13
Educational status				
Un able to write and read	134	38	34	29
Read and write only	161	45	70	59
Primary and above	60	17	14	12
Family size				
One to three	207	58	48	40
Four to five	137	38	61	52
Six and above	11	3	9	7
Residence				
Rural	274	77	83	70
Urban	81	23	35	30
Income				
Up to 500 birr	68	19	25	21
501 to 1000 birr	207	58	78	66
1001 to 1500 birr	59	16	13	11
Above 1500 birr	21	6	2	2
Distance				
Up to 5 kms	80	22	34	29
6 up to 10 kms	40	11	14	12
11 kms and above	235	66	70	59
Occupation				
Merchant	60	17	25	21
Farmer	241	69	78	66
Daily laborers	28	8	7	6

#### Medical and clinical characteristics of the respondents

From the study participants, 187 (52%) of controls were in WHO stage II while 78 (66%) of cases were in WHO stage III and IV. Two hundred ten (44.4%) cases and controls were clinically improved after starting their ART treatment. Three hundred twenty-three (91%) of controls and 88 (74%) of cases were adherent to their chronic HIV/AIDS care follow up. In addition, 117 (33%) controls and 74 (63%) cases were affected by opportunistic infections (Table 2)**.**

**Table 2:** Medical, clinical related characteristics of people living with HIV/AIDS, Shebel Berenta woreda, East Gojjam, Amhara region, North West Ethiopia, 2017

Variables	Controls (N = 355)		Cases (N = 118)	
	No	%	No	%
WHO stage				
WHO stage 1	60	17	9	7
WHCO stage 2	187	52	3	26
WHO stage 3 and 4	108	30	78	66
Current clinical condition				
Improved	307	86	99	84
Not improved	48	15	19	16
Current or past eating problems				
Yes	118	33	92	78
No	237	67	36	30
Health education				
Yes	331	93	97	82
No	24	7	20	5
Disclosure of ART status				
Yes	298	84	107	91
No	57	16	11	9
Duration on ART				
1 up 3 years	187	53	19	16
4 up to 6 years	131	37	51	43
7 up to 9 years	25	7	41	35
10 years and above	12	3	7	6
Interruption of treatment				
Yes	32	9	30	25
No	323	91	88	74
Current or past OI				
Yes	117	33	74	63
No	238	67	43	36

#### Nutritional related characteristics of the respondents

From the study participants, 270 (76%) controls and 20 (17%) cases changed their feeding style after knowing their ART status with quantity (70%), quality (16%) and frequency (14%) of changes.

#### Determinants of chronic energy deficiency

After controlling the cofounders, multivariable analysis result shows that WHO clinical stage, feeding style change, baseline CD4 cell count, family size, interruption of treatment, residence and monthly income were significantly determinant factors of Chronic energy deficiency in people living with HIV/IDS.

Those patients who were on clinical WHO stage I were 28.5% times more likely to develop CED [AOR: 0.285]. Feeding style changes during their treatment time were 7.5% times less likely develop CED [AOR: 0.075]. Those individuals who interrupted their treatment were 2.28 times more likely to be chronically energy deficient [AOR: 2.276]. The respondents who have CD4 count less than 200/mm3 and treatment interruption were 13.398 times more likely to develop CEM [AOR: 13.398] and 2.276 times to develop CEM [AOR: 2.276] respectively. Residents of rural areas were 37.6% times less likely to develop CEM than urban dweller [AOR: 0.38]. Patients who had an income level ≤ 500 birr were 6.91 times more likely to develop CEM than others [AOR: 6.91] (Table 3)**.**

**Table 3:** The bivariate and multivariable logistic regression analysis showing factors associated with Chronic Energy Deficiency in peoples living with HIV/AIDS, Shebel Berenta woreda, East Gojjam, Amhara region, North West Ethiopia, 2017

Variables	BMI < 18.5 kg/m^2^cases		BMI ≥ 18.5 kg//m^2^ controls		COR at 95% CI	AOR at 95% CI	p-value
	No	%	No	%			
WHO stage							
WHO stage 1	9	7	60	17	0.208 (0.097, 0.444)	0.285 (0.100, 0.810)	0.019
WHCO stage 2	31	26	187	52	0.230 (0.142, 0.371)	0.409 (0.205, 0.816)	0.011
WHO Stage 3	78	66	108	30	1	1	
Feeding style change							
Yes	20	17	270	76	0.060 (0.035, 0.105)	0.075 (0.038, 0.150)	0.000
No	98	83	85	24	1	1	
CD4 cell count							
Up 200 cells/mm^3^	61	53	73	20	19.19 (7.36, 35.593)	13.398 (4.83, 37.19)	0.000
201 up to 499 cells/mm^3^	49	42	127	36	7.475 (3.415, 16.362)	7.95 (2.982, 21.199)	0.019
500 and above	8	7	155	44	1	1	
Family size							
One to three	207	58	48	40	0.283 (0.111, 0.722)	0.114 (0.027, 0.475)	0.003
Four to five	137	38	61	52	0.544 (0.214, 1.381)		
Six and above	11	3	9	7	1	1	
Interruption of treatment							
Yes	32	9	30	25	3.441 (1.983, 5.970)	2.276 (1.017, 5.094)	0.045
No	323	91	88	74	1	1	
Monthly family income							
Up to 500 birr	68	19	25	21	3.860 (0.843, 17.670)	6.91 (1.069, 44.623)	0.042
501 to 1000 birr	207	58	78	66	3.957 (0.906, 17.270)		
1001 to 1500 birr	59	16	13	11	2.314 (0.481, 11.118)		
Above 1500 birr	21	6	2	2	1	1	
Residence							
Rural	83	70	274	70	0.701 (0.440, 1.118)	0.376 (0.171, 0.826)	0.015
Urban	35	30	81	30	1	1	

### Discussion

The aim of this study was to assess determinants of the chronic energy deficiency of adults LHIV. PLHIV who started their ART treatment during clinical WHO stage I were 71.5% less likely to become chronic energy deficient. This finding is in line with studies done in different parts of Ethiopia: in Addis Ababa [10], Humera hospital [11], Dilla [12] and Hosanna town [13]. This may be because of the low occurrence of opportunistic infections during the early stage of HIV/AIDS, which are the immediate causes of under nutrition. But the finding is different from the study conducted in Nekemte and Gondar Hospitals, Ethiopia [11, 14, 15]. This variation may be due to the difference in study design, study periods and study area.

PLHIV who changed their feeding style were, 92.5% less likely to have chronic energy deficiency than counterparts. This finding is consistent with the findings of the studies conducted in AA [16], Humera hospital [11], Hosanna town [13] and Metema hospital [17]. However, studies conducted in Nekemte [14] and Gondar hospitals [15] have contrastive result with the current study. This variation may be due to the difference in study design, study period, the facility sets up and differences in sample size.

This study revealed that PLHIV/AIDS who started their ART with low baseline CD4 cell count were 13.389 times more likely to have a chronic energy deficiency. This might be as CD4 cell increases, there might be reduction of viral load and it leads to reduction of opportunistic infection which caused under nutrition. This finding is in line with studies conducted in the United States [18] and public hospital of Addis Ababa [10], but it disagrees with the study conducted in the Humera Hospital [11]. This variation may be due to the difference in study design, study period, facility setup, different in sample size, and participants’ health seeking behaviors.

PLHIV who lived in rural areas were 62.4% less likely to be chronically energy deficient than those who lived in urban areas. Most urban poor people are more vulnerable to HIV/AIDS. Though this finding is supported by the study conducted in Dares-Salaam [19] but inconsistent with the finding of the study conducted in Butajira hospital [20].

Alike finding of the study conducted in Gondar referral hospital [15], the current study revealed that people living with HIV/AIDS and who had low monthly family income were 6.91 times more likely to be chronically energy deficient. The study in Dilla contrarily stated that moderately poor economic condition has protective role the deficiency [12].

The study found that interruption of ART treatment resulted in a chronic energy deficiency. People living with HIV/AIDS who interrupted their ART treatment were 2.276 times more likely to be chronically energy deficient than their counterparts. The finding is in line with the study conducted in Northern Ethiopia [21] and Nekemte referral hospital [14]. But it disagrees with the study conducted in selected public Hospital of Addis Ababa [10] and Humera Hospital [11]. The variation may be due to the study design, place, and study periods may attribute to such result differences.

People living with HIV/AIDS and who have less than four family size were 88.6% times less likely to have chronic energy deficiency than others. This may be due to the households’ ability to afford food expenses if there is limited family member.

Clinical WHO stage I, residence, feeding style change and family size decreased chronic energy deficiency status, but, baseline CD4 less than 200/mm^3^ and low income increased the risk of chronic energy deficiency. Therefore, health professionals at different level should strengthen ART service to initiate HAART as early as possible and promote family planning to limit the family size. Health care providers should strongly counsel HIV/AIDS patients about their way of feeding. Clients should engage in income generating activities to increase the monthly income.

## Limitation of the study

There might be selection bias and misclassification bias for some independent variables and since this study was institution based, generalizing to the general population difficult.

## Data Availability

The datasets used and/or analyzed during the current study are available from the corresponding author. The data will not be shared in order to preserve participant anonymity.

## Abbreviations

AIDS: acquired immune deficiency syndrome
AOR: adjusted odds ratio
ART: anti-retroviral therapy
BMI: body mass index
CD4: cluster of differentiation 4
CI: confidence interval
DMU: Debre Markos University
PLWHIV: people living with HIV AIDS
SD: standard deviations
TB: tuberculosis
WHO: World Health Organization
